# Clinical characteristics of pediatric patients hospitalized with community-acquired pneumonia and cytomegalovirus DNA detected in bronchoalveolar lavage fluid

**DOI:** 10.3389/fped.2024.1407174

**Published:** 2024-07-24

**Authors:** Xinyu Wang, Yanhong Lu, Feng Chen, Linan Ruan, Lingtong Gu, Ting Wang, Heting Dong, Yuqing Wang, Chuangli Hao, Li Huang, Yongdong Yan, Huiming Sun, Zhengrong Chen

**Affiliations:** ^1^ Clinical Pediatrics School, Soochow University, Suzhou, Jiangsu, China; ^2^ Department of Respiratory Medicine, Children’s Hospital of Soochow University, Suzhou, Jiangsu, China; ^3^ Department of Respiratory Medicine, Luodian Hospital, Shanghai, China

**Keywords:** cytomegalovirus, blood, urine, community-acquired pneumonia, immunocompetent

## Abstract

**Background:**

This study aimed to investigate the clinical characteristics of pediatric patients hospitalized with community-acquired pneumonia (CAP) and concomitant cytomegalovirus (CMV) infection.

**Methods:**

This cross-sectional study enrolled consecutive pediatric patients admitted with CAP who tested positive for CMV DNA in bronchoalveolar lavage fluid (BALF). Flexible fiberoptic bronchoscopy was performed when routine treatment for CAP proved ineffective. The study participants were further stratified into two groups based on CMV serological test results: recent CMV infection group and CMV replication group. Clinical characteristics were compared between these two groups.

**Results:**

Among 124 patients aged 1–11 months included in this study, 80 (64.5%) patients were categorized as having recent CMV infection, and 44 (35.5%) tested positive for CMV replication. Co-infection with other pathogens was detected more frequently in the CMV replication group (*n *= 29, 65.9%) than in the recent CMV infection group (*n *= 35, 43.7%; *P *= 0.018). Patients with recent CMV infection were younger and exhibited higher levels of alanine transaminase (ALT) and aspartate aminotransferase compared to those with CMV replication (all *P *< 0.05). Multivariable regression analysis showed age was independently associated with recent CMV infection (odds ratio [OR], 0.707; 95% confidence interval [CI], 0.586–0.853; *P *< 0.001). Notably, receiver operating characteristic curve analysis showed that a CMV PCR level of 3,840 copies/ml in blood samples had a sensitivity of 34.7% and specificity of 90.0% for diagnosis of recent CMV infection with an area under the curve (AUC) of 0.625 (95% CI: 0.513–0.736, *P *= 0.048). A CMV PCR level of 6,375 copies/ml in urine samples had a sensitivity of 77.1% and specificity of 61.5% for diagnosis of recent CMV infection with an AUC of 0.695 (95% CI: 0.531–0.858, *P *= 0.04). Furthermore, multivariate linear regression analysis revealed that the blood CMV DNA copy number was associated with ALT (*B* = 0.001; *P *< 0.001).

**Conclusions:**

The CMV DNA copy numbers in blood and urine could serve as discriminatory markers between recent CMV infection and CMV replication. Measuring CMV DNA levels in blood may be an effective method for monitoring liver function impairment in pediatric patients presenting with CAP and concurrent CMV infection.

## Introduction

Cytomegalovirus (CMV) is common virus, and its prevalence increases with age. It is reported that almost 85% of infants are infected within 1 year after birth (1, 2). After the initial infection, CMV establishes life-long persistence, alternating between latent phases and periods of active infection (3). The interplay between CMV and host immunity may play a role in determining the course of the disease (4, 5).

CMV infection can cause a variety of diseases, including pneumonitis, hepatitis, bacterial superinfection, colitis, encephalitis, and myocarditis (6). Among immunocompetent children hospitalized with community-acquired pneumonia (CAP), CMV is one of the most frequently identified viruses (7). The lungs are an important site for CMV reactivation. Typically, bronchoalveolar lavage fluid (BALF) is collected as a specimen to detect CMV for diagnosis of CMV lung infection (8–10). However, the presence of CMV DNA in BALF may not always be associated with acute CMV pulmonary illness, since CMV can potentially reactivate due to airway inflammation (8, 11). To date, investigations into the clinical characteristics of immunocompetent pediatric CAP patients who test positive for CMV DNA in BALF have been limited.

Currently, there are no anti-CMV treatment guidelines for immunocompetent pediatric patients. CMV IgM positivity is one of the clinical indicators for the diagnosis of CMV pneumonia in immunocompetent patients (12), and patients with severe CMV-related pneumonitis usually need antiviral treatment (13). In addition, establishing a link between CMV detection in BALF, urine, or blood samples and specific symptoms is of great medical importance, as it could also indicate the need for antiviral therapy. In the present study, patients with CMV DNA detected in BALF were categorized into two groups, the recent CMV infection group and CMV replication group, based on CMV serological testing, for comparison of clinical characteristics between these two groups. Additionally, we investigated the association between CMV DNA copy number in different types of samples and the levels of liver enzymes in CAP patients.

## Methods

### Patients

This cross-sectional study was conducted at the Children's Hospital of Soochow University, a tertiary teaching hospital in Suzhou, Jiangsu Province, China, from January 2019 to December 2022. During the study period, consecutive immunocompetent pediatric patients under 1 year of age who were admitted with CAP and tested positive for CMV DNA in BALF were screened. This age cutoff was chosen because host immunity is a crucial factor in diagnosing lung CMV infection (14), and young children with immature immune systems may be more susceptible to CMV infection. Flexible fiberoptic bronchoscopy was performed when routine treatment for CAP was ineffective, as the detection of CMV in BALF raised suspicion of it being a possible etiologic agent of CAP. CAP was diagnosed according to the Chinese Society of Pediatrics guidelines (15). The exclusion criteria were: age <28 days, premature birth or birthweight <2,500 g, blood transfusion within the previous 2 weeks, congenital or acquired immunodeficiencies, treatment with certain anti-CMV drugs within the previous 2 months (including ganciclovir, acyclovir, or valganciclovir), rheumatoid arthritis, and history of human herpes virus-6 or Epstein-Barr virus infection. The study was approved by the Ethics Committee of the Children's Hospital of Soochow University (2023CS162).

### Study protocol

The following data were collected: (1) demographic data, including sex and age; (2) clinical data, including presence of fever, wheezing, requirement of supplemental oxygen, admission to the pediatric intensive care unit (PICU), requirement of mechanical ventilation, and requirement of Ganciclovir treatment; (3) laboratory data, including peripheral leukocyte count, neutrophil percentage, hemoglobin, platelet count, C-reactive protein, alanine aminotransferase (ALT), aspartate aminotransferase (AST), and BALF cell profile; (4) presence of serum IgM and IgG anti-CMV as determined by magnetic microparticle chemiluminescence method using a CMV antibody detection kit (Autobio Diagnostics Co., Ltd. Zhengzhou, China), following the manufacturer's instructions, with CMV IgM titers ≥8 AU/ml and CMV IgG titers ≥10 AU/ml considered as positive; (5) CMV DNA in BALF, whole blood, and urine as detected by using the CMV Nucleic Acid Test kit (Sansure Biotech Co., Ltd. Hunan, China), following the manufacturer's instructions, with a detection limit >400 copies/ml (16); (6) differential cell counts in BALF as determined using a modified version of Wright-Giemsa staining (Baso Diagnostics Inc., Zhuhai, China), as previously described (16); (7) 11 common respiratory pathogens, including influenza A and influenza B viruses, human bocavirus, adenovirus, human rhinovirus, parainfluenza virus, coronaviruses, respiratory syncytial virus, human metapneumovirus, *Mycoplasma pneumoniae* and *Chlamydophila pneumoniae* screened in nasopharyngeal swab samples using a respiratory pathogen multiples detection kit (Health Gene Tech., Ningbo, China) (17); (8) bacteria detection in BALF, with growth >10^4^ cfu/ml considered significant (18); and (9) treatment with ganciclovir therapy (10 mg/kg/day) after admission as decided by the attending physician (19).

The laboratory tests were conducted within 6 h after admission. Detection of the 11 common respiratory pathogens was performed within 24 h after admission. Flexible fiberoptic bronchoscopy was conducted in adherence to recommendations from the China Respiratory Society (15). Whole blood CMV PCR and urine CMV PCR was performed for patients who tested positive for CMV DNA in BALF.

### Study definitions

The presence of measurable CMV DNA in BALF combined with a positive CMV IgM titer or a 4-fold increase in the CMV IgG titer was defined as recent CMV infection (19). Conversely, detection of measurable CMV DNA in BALF without a positive CMV IgM titer or a 4-fold increase in the CMV IgG titer was defined as CMV replication (20).

### Statistical analysis

Statistical analyses were conducted using SPSS (version 21). The normality of numerical data was determined by the Shapiro-Wilk test. Non-normally distributed data are presented as median [interquartile range (IQR)]. Between-group comparisons were performed using the Kruskal–Wallis test or Mann–Whitney *U*-test, as appropriate. The Chi-squared test was used to assess differences in categorical variables. Variables with a *P* value < 0.05 on univariate analysis were considered for inclusion in the multivariate logistic regression models. The optimal CMV load cutoff for recent CMV infection was determined by receiver operating characteristic (ROC) curve analysis. The correlations between CMV copy number and ALT level were determined using the Spearman correlation analysis. Multiple linear regression analysis was conducted to identify factors independently associated with the ALT level. A value of *P *< 0.05 was considered statistically significant.

## Results

### Demographic characteristics

Between January 2019 and December 2022, a total of 124 patients, aged from 1 to 11 months [median, 3.0 months (IQR 2.0–6.0 months)], were admitted with CAP and tested positive for CMV DNA in BALF. Of these patients, 80 (64.5%, 80/124) were categorized as having recent CMV infection, and 44 (35.5%, 44/124) were classified as cases of CMV replication.

### Co-infections

**Figure 1:**
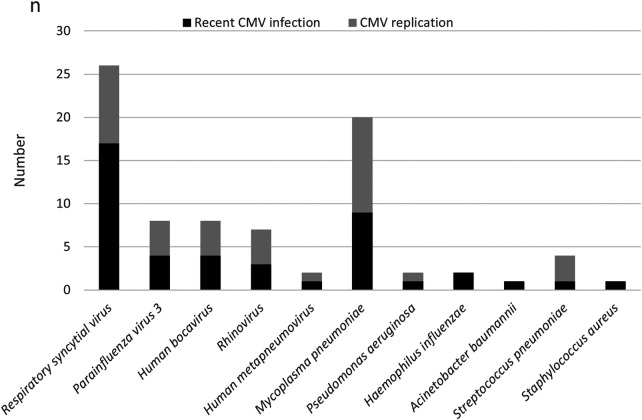
Co-infections in 124 patients hospitalized with CAP and CMV infection.

In 51.6% of the cases (64/124 patients), a secondary pathogen was detected. Co-infection was more frequently detected in the CMV replication group (*n* = 29, 29/44) than in the recent CMV infection group (*n* = 35, 35/80; 65.9% vs. 43.7%, *P *= 0.018). Respiratory syncytial virus (21.0%, 26/124) and *M. pneumoniae* (16.1%, 20/124) were the most commonly detected co-infecting pathogens (Figure 1).

### Comparison of clinical characteristics

The clinical characteristics of patients in the two groups are presented in Table 1. In comparison with the CMV replication group, the recent CMV infection group had a younger age (*P* < 0.001), lower prevalence of wheezing (*P *= 0.027), and lower percentage of patients who required supplemental oxygen (*P* = 0.019). Regarding laboratory findings, patients with recent CMV infection exhibited a lower hemoglobin level but higher ALT and AST levels than patients with CMV replication (all *P* < 0.05).

**Table 1:** Comparison of the clinical characteristics of patients with recent CMV infection and CMV replication.

Parameter	Recent CMV infection group (*n* = 80)	CMV replication group (*n* = 44)	*P*
General characteristics			
Male	51 (61.7)	34 (77.3)	0.121
Age, months	2.5 (2.0–4.0)	5.0 (3.0–7.7)	<0.001
Clinical signs and symptoms			
Fever	26 (32.5)	16 (36.4)	0.664
Wheezing	48 (60.0)	35 (79.5)	0.027
Disease severity			
Requirement for supplemental oxygen	23 (28.7)	22 (50.0)	0.019
PICU admission	11 (13.7)	8 (16.0)	0.512
Mechanical ventilation	6 (7.5)	6 (13.6)	0.343
Laboratory findings			
Peripheral leukocyte count, 10^9^/L	11.8 (8.8–14.8)	12.6 (9.0–16.4)	0.465
Neutrophil count, %	25.0 (17.9–35.5)	24.2 (15.5–50.9)	0.473
Hemoglobin, g/L	111.0 (103.0–117.0)	115.0 (107.0–126.0)	0.004
Platelet number, 10^9^/L	394.0 (325.5–475.0)	423.0 (351.0–531.0)	0.110
C-reactive protein, mg/dl	1.4 (0.7–7.0)	1.1 (0.2–7.0)	0.251
Alanine transaminase, U/L	38.1 (24.3–80.2)	27.4 (20.3–36.1)	0.004
Aspartate aminotransferase, U/L	54.9 (42.4–92.6)	46.8 (38.1–56.9)	0.011
Bronchoalveolar lavage fluid cell profile			
Neutrophil, %	25.0 (10.0–70.0)	45.0 (10.0–70.0)	0.413
Alveolar macrophages, %	70.0 (22.0–87.5)	55.0 (25.0–85.0)	0.511
Lymphocytes, %	2.0 (1.0–5.0)	2.0 (0–6.0)	0.781
Eosinophils, %	0 (0–0)	0 (0–0)	0.410

Data are presented as median (IQR) or *n* (%), unless otherwise indicated.

The following characteristics did not differ significantly between the two groups: gender ratio, rate of fever, rate of PICU admission, mechanical ventilation rate, peripheral leukocyte count, neutrophil percentage, platelet count, level of C-reactive protein, and white blood cell profile (including lymphocyte, alveolar macrophage, eosinophil and neutrophil percentages) in BALF.

### Risk factors for recent CMV infection

We next performed multivariate logistic regression analysis to identify risk factors associated with recent CMV infection. Age (odds ratio [OR], 0.707; 95% confidence interval [CI], 0.586–0.853; *P *< 0.001) was identified as a significant predictor of recent CMV infection.

### Comparison of CMV DNA copy number

**Figure 2:**
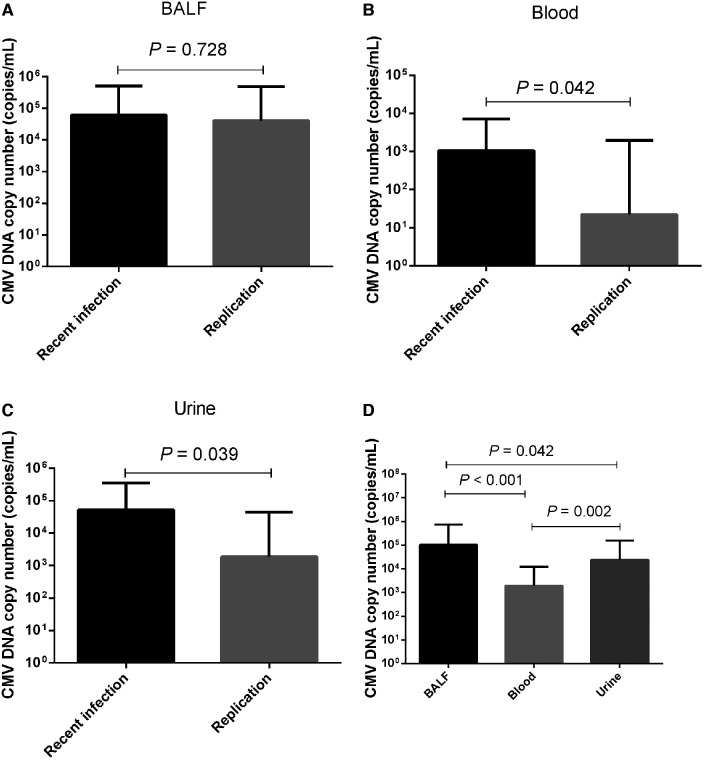
Comparison of CMV DNA copy numbers. Comparison of CMV DNA copy numbers in (**A**) BALF, (**B**) blood, and (**C**) urine between patients with recent CMV infection and CMV replication. (**D**) CMV DNA copy numbers in BALF, blood, and urine.

No significant difference in the median BALF CMV DNA copy number was detected between the recent CMV infection and CMV replication groups [median 61,300.0 copies/ml (IQR 7,772.5–512,000.0 copies/ml)] vs. 40,950.0 copies/ml [9,507.5–488,750.0 copies/ml], *P *= 0.728; Figure 2A). Among the 80 patients with recent CMV infection, blood CMV PCR was performed for 72 patients, and 47 (65.3%) tested positive. Similarly, among the 44 patients with CMV replication, blood CMV PCR was performed for 30 patients, and 15 (50.0%) tested positive. The blood CMV PCR positivity rate did not differ significantly between the two groups (65.3% vs. 50.0%, *P* = 0.150). The median CMV DNA copy number in blood was higher in patients with recent CMV infection than in patients with CMV replication (median 1,065.0 copies/ml [IQR 0–7,223.7 copies/ml] vs. 250.0 copies/ml [0–1,950.0 copies/ml], *P *= 0.042; Figure 2B).

Among the 80 patients with recent CMV infection, urine CMV PCR was performed for 35 patients, and 29 (82.8%) tested positive. For the 44 patients with CMV replication, urine CMV PCR was performed for 13 patients, and 9 (69.2%) tested positive. The urine CMV PCR positivity rate did not differ significantly between the two groups (82.8% vs. 69.2%, *P *= 0.425). The median CMV DNA copy number in urine was higher in patients with recent CMV infection than in patients with CMV replication (median 52,000.0 copies/ml [IQR 7,750.0–353,000.0 copies/ml] vs. 1,870.0 copies/ml [0–45,700.0 copies/ml], *P *= 0.039; Figure 2C). Out of all 124 CMV-positive patients, BALF, blood, and urine CMV PCR were simultaneously performed for 43 patients. The median CMV DNA copy number in BALF was higher than in urine (median 104,000.0 copies/ml [IQR 8,280.0–35,000.0 copies/ml] vs. 23,900.0 copies/ml [856.0–155,000.0 copies/ml], *P *= 0.042), as well as higher than in blood (median 104,000.0 copies/ml [IQR 8,280.0–735,000.0 copies/ml] vs. 1,950.0 copies/ml [0–12,200.0 copies/ml], *P *< 0.001). The median CMV DNA copy number was higher in urine than in blood (median 23,900.0 copies/ml [IQR 856.0–155,000.0 copies/ml] vs. 1,950.0 copies/ml [0–12,200.0 copies/ml], *P *= 0.002; Figure 2D).

### ROC curve analysis

**Figure 3:**
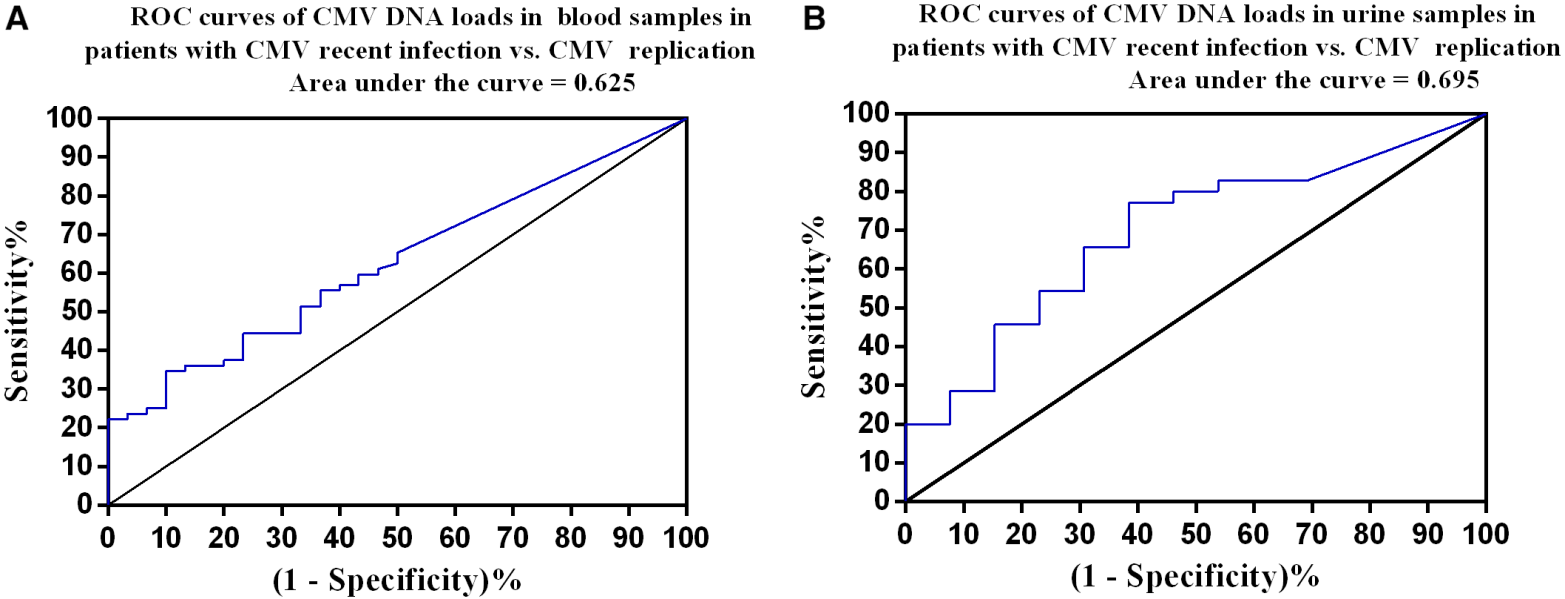
Receiver operating characteristic curves. (**A**) Patients with recent CMV infection (*n* = 72) vs. CMV replication (*n* = 30). The optimal cutoff was 3,840 copies/ml in blood samples, with a sensitivity of 34.7% and specificity of 90%. (**B**) Patients with recent CMV infection (*n* = 35) vs. CMV replication (*n* = 13). The optimal cutoff was 6,375 copies/ml in urine samples, with a sensitivity of 77.1% and specificity of 61.5%.

We constructed ROC curves for CMA DNA loads in blood and urine samples to determine thresholds with optimal sensitivity and specificity for recent CMV infection. ROC curve analysis showed that a CMV PCR level of 3,840 copies/ml in blood samples had a sensitivity of 34.7% and specificity of 90.0% for diagnosis of recent CMV infection with an area under the curve (AUC) of 0.625 (95% CI: 0.513–0.736, *P *= 0.048; Figure 3A). A CMV PCR level of 6,375 copies/ml in urine samples had a sensitivity of 77.1% and specificity of 61.5% for diagnosis of recent CMV infection with an AUC of 0.695 (95% CI: 0.531–0.858, *P *= 0.04; Figure 3B).

### Association between CMV DNA copy number and liver enzyme levels

**Figure 4:**
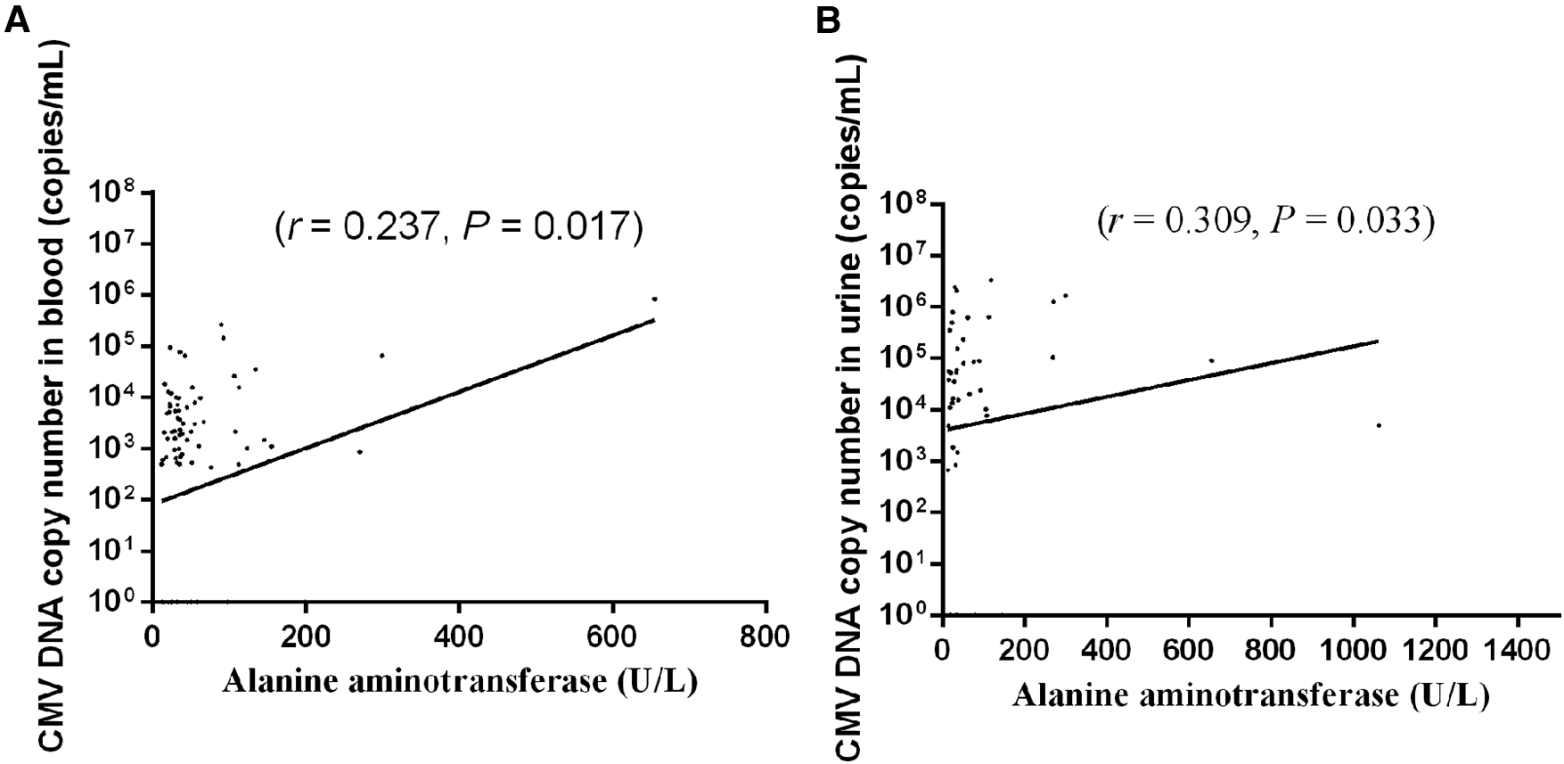
Association between CMV DNA copy number in (**A**) blood and (**B**) urine and liver enzyme level. Each dot indicates an individual patient.

The associations between the CMV DNA copy number in different samples (BALF, blood, and urine) and liver enzyme levels (ALT, AST) were examined. The results showed that the blood CMV DNA copy number [median 733.5 copies/ml (IQR 0–5,260.0 copies/ml)] exhibited a positive correlation with the ALT level (*r *= 0.237, *P *= 0.017; Figure 4A). The urine CMV DNA copy number [median 22,150.0 copies/ml (IQR 1,012.0–142,750.0 copies/ml)] was positively correlated with the ALT level (*r *= 0.309, *P *= 0.033; Figure 4B). The BALF CMV DNA copy number [median 50,600.0 copies/ml (IQR 8,265.0–512,000.0 copies/ml)] was not correlated with the ALT level (*r *= 0.029, *P *= 0.747).

In addition, multivariate linear regression analysis revealed that the blood CMV DNA copy number was associated with the ALT level (*B* = 0.001; *P *< 0.001).

### Treatment with ganciclovir therapy

Among the 80 patients with recent CMV infection, 43 (53.7%) received ganciclovir therapy (10 mg/kg/day). In contrast, of the 44 patients with CMV replication, 10 (22.7%) received ganciclovir therapy (10 mg/kg/day). The utilization rate of ganciclovir was notably higher in patients with recent CMV infection than in those with CMV replication (53.7% vs. 22.7%, *P *= 0.001). All patients recovered regardless of treatment.

## Discussion

Deciphering the implications of CMV DNA detection in BALF by PCR poses a challenge for pediatric practitioners, and standardized clinical criteria for immunocompetent patients are lacking. In the present study, immunocompetent pediatric patients under 1 year old, who were admitted with CAP and tested positive for CMV DNA in BALF, were further categorized according to the results of CMV serological testing. It was surprising that 64.5% of the patients had CMV recent infection. Age was identified as a significant predictor of recent CMV infection. Consequently, pediatricians should exercise vigilance regarding the possibility of recent CMV infection when managing young patients hospitalized for CAP who have tested positive for CMV DNA in BALF.

In the present study, a secondary pathogen was identified more frequently among cases categorized as CMV replication than among those categorized as recent CMV infection. This observation might not necessarily suggest a causative role for CMV in patients with CMV replication. In addition, the rate of ganciclovir use was higher in patients with recent CMV infection and no detection of a secondary pathogen (*n* = 30, 30/45) than in those with recent CMV infection and detection of a secondary pathogen (*n* = 13, 13/35; 66.7% vs. 37.1%, *P *= 0.009), which might suggest that CMV was usually considered the etiology of cases in which no secondary pathogen was detected. Furthermore, the recent CMV infection group had a lower rate of wheezing than the CMV replication group. This could be attributed to the finding that the most commonly detected pathogens were respiratory syncytial virus and *M. pneumoniae*, both of which are known to increase susceptibility to wheezing in young children (21, 22).

In our study, the percentage of patients who required supplemental oxygen was lower in the recent CMV infection group than in the CMV replication group. The reason might be that CMV infection in immunocompetent individuals rarely leads to severe illness (23). Furthermore, patients with recent CMV infection had a lower hemoglobin lever than patients with CMV replication, which may be linked to physiologic anemia. Additionally, patients with recent CMV infection had higher ALT and AST levels than patients with CMV replication, indicating that recent CMV infection is more commonly associated with elevation of hepatic aminotransferase levels. However, the ALT and AST levels were not significantly elevated, aligning with the findings from a previous study (24). Notably, the levels of hepatic aminotransferases in cases of CMV infection are generally lower than those in cases of hepatitis caused by hepatitis viruses.

Our results showed that the median BALF CMV DNA copy number did not differ significantly between patients with recent CMV infection and CMV replication. This suggests that the BALF CMV DNA copy number might not be a discriminatory marker for recent CMV infection vs. CMV replication. However, the median CMV DNA copy numbers in blood and urine were higher in patients with recent CMV infection than in patients with CMV replication. ROC curves were constructed and used to determine a cut-off value of 3,840 copies/ml for the CMV PCR level in blood samples (sensitivity: 34.7% and specificity: 90.0%) and a cut-off value of 6,375 copies/ml for the CMV PCR level in urine samples (sensitivity: 77.1% and specificity: 61.5%) for distinguishing recent CMV infection from CMV replication. These results indicate that the CMV DNA copy numbers in blood and urine could serve as discriminators between recent CMV infection and CMV replication. However, both had relatively low AUC values, which may indicate poor diagnostic performance (25). In addition, the median CMV DNA copy number was higher in BALF samples than in blood and urine samples, which might support the concept of a compartmentalization and spill-over effect in the pathogenic response. Thus, having CMV disease in the lungs should theoretically lead to higher CMV loads within the respiratory system, with a spill-over effect extending into the bloodstream when CMV replication cannot be effectively controlled within the lungs (26, 27).

Our results revealed a positive correlation between the blood CMV DNA copy number and ALT level, similar to the results of a previous study (28). This suggests that the level of blood CMV DNA could potentially serve as a monitoring parameter for CMV-associated infantile hepatitis. However, the *r* value was low, indicating a weak correlation.

This study has several limitations. Firstly, we did not dynamically monitor CMV loads in blood, urine, and BALF samples, which could provide better insight into the correlation between virus load and clinical characteristics and is warranted in further investigations. Secondly, while 10 (22.7%) patients with CMV replication recovered after ganciclovir therapy, the number with recent CMV infection may have been underestimated due to serological testing limitations (23). Future multicenter, randomized clinical trials are needed to determine the indications for ganciclovir as well as its efficacy in immunocompetent patients. Thirdly, our study only included patients over 1 month old, limiting the generalizability of the results to younger infants who require separate evaluation. Fourthly, PCR for CMV in BALF, blood, and urine samples simultaneously was not performed for all patients. Comparison of the clinical characteristics of children with recent CMV infection and CMV replication based on whether blood and urinary CMV tests had been performed showed that patients who underwent urine CMV testing were younger than those who did not among patients with recent CMV infection (*P *= 0.040), and the percent of lymphocytes in BALF was lower in patients for whom urine CMV testing was performed than in those for whom it was not (*P *= 0.025) (Supplementary Tables S1–S4). Thus, population bias might exist, and future large-sample clinical studies are needed. Fifthly, we did not set a control group of children with no respiratory symptoms; thus, the percentage of children with recent CMV infection among those with no respiratory symptoms is unknown. However, performing flexible fiberoptic bronchoscopy in children with no respiratory symptoms is unethical. Finally, we did not employ the test for 11 respiratory viruses in BALF due to the potential for nasopharyngeal swabs to partially represent lung pathogens (29), the high cost of the test, and patient reluctance regarding repeat testing. However, we aim to include this analysis in future studies when feasible and permitted by patients' guardians.

In conclusion, it is imperative for pediatricians to maintain a high degree of suspicion for recent CMV infection when managing young patients hospitalized with CAP and a history of CMV DNA detection in BALF. Furthermore, the CMV DNA copy numbers in blood and urine could serve as discriminatory markers between recent CMV infection and active CMV replication. Importantly, testing for CMV DNA levels in blood could prove valuable for monitoring liver function impairment in pediatric patients presenting with CAP and concurrent CMV infection.

## Data Availability

The raw data supporting the conclusions of this article will be made available by the authors, without undue reservation.
