# Supplementary material for: Clinical characteristics of pediatric patients hospitalized with community-acquired pneumonia and cytomegalovirus DNA detected in bronchoalveolar lavage fluid
Source: Front Pediatr. 2024 Jul 24;12:1407174. doi: 10.3389/fped.2024.1407174 (PMC11303221; doi:10.3389/fped.2024.1407174)
Supplement: Supplementary file 1 [file Table1.docx]

Table S1. Comparison of the clinical characteristics of patients with CMV recent infection for whom blood CMV testing was or was not performed.

| Parameter | Patients who underwent blood CMV testing (n=72) | Patients who did not undergo blood CMV testing (n=8) | *P* |
| --- | --- | --- | --- |
| General characteristics | |  |  |
| Male | 47 (65.3) | 4 (50.0) | 0.394 |
| Age | 3.0 (2.0-4.7) | 2.0 (2.0-2.7) | 0.206 |
| Clinical signs and symptoms | |  |  |
| Fever | 25 (34.7) | 1 (12.5) | 0.203 |
| Wheezing | 43 (59.7) | 5 (62.5) | 0.879 |
| Disease severity |  |  |  |
| Requirement for supplemental oxygen | 21 (29.2) | 2 (25.0) | 0.805 |
| PICU admission | 10 (13.9) | 1 (12.5) | 0.914 |
| Mechanical ventilation | 5 (6.9) | 1 (12.5) | 0.571 |
| Laboratory findings | |  |  |
| Peripheral leukocyte count, 10^9^/L | 11.8 (8.7-14.7) | 11.8 (8.9-15.6) | 0.974 |
| Neutrophil count, % | 25.5 (16.9-36.5) | 23.5 (18.7-27.7) | 0.480 |
| Hemoglobin, g/L | 113.0 (103.5-118.0) | 103.5 (100.2-108.0) | 0.093 |
| Platelet count, 10^9^/L | 394.0 (333.5-473.2) | 386.5 (242.7-533.0) | 0.736 |
| C-reactive protein, mg/dL | 0.9 (0-3.1) | 1.6 (0.06-2.6) | 0.812 |
| Alanine transaminase, U/L | 38.1 (24.3-78.9) | 56.3 (21.4-108.8) | 0.637 |
| Aspartate aminotransferase, U/L | 53.0 (41.7-92.1) | 68.4 (52.2-150.5) | 0.199 |
| Bronchoalveolar lavage fluid cell profile | | |  |
| Neutrophils, % | 25.0 (10.0-74.0) | 16.0 (4.0-58.0) | 0.822 |
| Alveolar macrophages, % | 70.0 (20.0-87.0) | 70.5 (38.0-90.0) | 0.50 |
| Lymphocytes, % | 2.0 (1.0-5.0) | 1.0 (0-4.0) | 0.163 |
| Eosinophils, % | 0 (0-0) | 0 (0-0) | 0.528 |

Data are presented as median (IQR) or n (%), unless otherwise indicated.
