# Supplementary material for: Clinical characteristics of pediatric patients hospitalized with community-acquired pneumonia and cytomegalovirus DNA detected in bronchoalveolar lavage fluid
Source: Front Pediatr. 2024 Jul 24;12:1407174. doi: 10.3389/fped.2024.1407174 (PMC11303221; doi:10.3389/fped.2024.1407174)
Supplement: Supplementary file 2 [file Table2.docx]

Table S2. Comparison of the clinical characteristics of patients with CMV recent infection for whom urine CMV testing was or was not performed.

| Parameter | Patients who underwent urine CMV testing (n=35) | Patients who did not undergo urine CMV testing (n=45) | *P* |
| --- | --- | --- | --- |
| General characteristics |  |  |  |
| Male | 25 (71.4) | 26 (57.8) | 0.208 |
| Age | 2.5 (2.0-4.0) | 5.0 (3.0-7.7) | 0.040 |
| Clinical signs and symptoms | |  |  |
| Fever | 12 (34.3) | 14 (31.1) | 0.764 |
| Wheezing | 21 (60.0) | 27 (60.0) | >0.999 |
| Disease severity |  |  |  |
| Requirement for supplemental oxygen | 10 (28.6) | 13 (28.9) | 0.975 |
| PICU admission | 6 (17.1) | 5 (11.1) | 0.437 |
| Mechanical ventilation | 3 (8.6) | 3 (6.7) | 0.748 |
| Laboratory findings |  |  |  |
| Peripheral leukocyte count, 10^9^/L | 11.8 (8.8-14.8) | 12.6 (9.0-16.4) | 0.464 |
| Neutrophil count, % | 25.0 (17.9-35.5) | 24.2 (15.5-50.9) | 0.194 |
| Hemoglobin, g/L | 111.0 (103.0-117.0) | 115.0 (107.0-126.0) | 0.382 |
| Platelet count, 10^9^/L | 394.0 (325.5-475.0) | 423.0 (351.0-531.0) | 0.383 |
| C-reactive protein, mg/dL | 1.4 (0.7-7.0) | 1.1 (0.2-7.0) | 0.738 |
| Alanine transaminase, U/L | 38.1 (24.3-80.2) | 27.4 (20.3-36.1) | 0.554 |
| Aspartate aminotransferase, U/L | 54.9 (42.4-92.6) | 46.8 (38.1-56.9) | 0.410 |
| Bronchoalveolar lavage fluid cell profile | |  |  |
| Neutrophils, % | 25.0 (10.0-70.0) | 45.0 (10.0-70.0) | 0.367 |
| Alveolar macrophages, % | 70.0 (22.0-87.5) | 55.0 (25.0-85.0) | 0.150 |
| Lymphocytes, % | 2.0 (1.0-5.0) | 2.0 (0-6.0) | 0.025 |
| Eosinophils, % | 0 (0-0) | 0 (0-0) | 0.448 |

Data are presented as median (IQR) or n (%), unless otherwise indicated.
