# Supplementary material for: Clinical characteristics of pediatric patients hospitalized with community-acquired pneumonia and cytomegalovirus DNA detected in bronchoalveolar lavage fluid
Source: Front Pediatr. 2024 Jul 24;12:1407174. doi: 10.3389/fped.2024.1407174 (PMC11303221; doi:10.3389/fped.2024.1407174)
Supplement: Supplementary file 3 [file Table3.docx]

Table S3. Comparison of the clinical characteristics of patients with CMV replication for whom blood CMV testing was or was not performed.

| Parameter | Patients who underwent blood CMV testing (n=30) | Patients who did not undergo blood CMV testing (n=14) | *P* |
| --- | --- | --- | --- |
| General characteristics |  |  |  |
| Male | 22 (73.3) | 12 (85.7) | 0.361 |
| Age | 5.0 (2.0-7.2) | 6.0 (3.0-8.2) | 0.323 |
| Clinical signs and symptoms | |  |  |
| Fever | 10 (33.3) | 6 (42.9) | 0.541 |
| Wheezing | 22 (73.3) | 13 (92.9) | 0.135 |
| Disease severity |  |  |  |
| Requirement for supplemental oxygen | 12 (40.0) | 4 (28.6) | 0.463 |
| PICU admission | 4 (13.3) | 4 (28.6) | 0.222 |
| Mechanical ventilation | 4 (13.3) | 2 (14.3) | 0.932 |
| Laboratory findings |  |  |  |
| Peripheral leukocyte count, 10^9^/L | 12.9 (8.3-16.4) | 12.4 (9.9-21.1) | 0.821 |
| Neutrophil count, % | 25.2 (15.8-54.8) | 24.1 (15.0-43.8) | 0.880 |
| Hemoglobin, g/L | 119.0 (110.0-128.0) | 113.5 (107.0-125.2) | 0.459 |
| Platelet count, 10^9^/L | 400.0 (329.5-542.0) | 436.5 (407.5-518.7) | 0.325 |
| C-reactive protein, mg/dL | 0.6 (0.08-2.8) | 0.3 (0-9.2) | 0.533 |
| Alanine transaminase, U/L | 28.3 (21.6-36.7) | 23.7 (19.4-35.1) | 0.378 |
| Aspartate aminotransferase, U/L | 50.0 (38.2-57.3) | 43.9 (35.4-54.5) | 0.406 |
| Bronchoalveolar lavage fluid cell profile | |  |  |
| Neutrophils, % | 38.0 (5.5-67.5) | 16.5 (8.7-59.0) | 0.607 |
| Alveolar macrophages, % | 52.5 (25.0-70.0) | 73.0 (31.5-89.5) | 0.410 |
| Lymphocytes, % | 3.0 (0.7-10.0) | 2.0 (0-4.0) | 0.158 |
| Eosinophils, % | 0 (0-0) | 0 (0-0) | 0.925 |

Data are presented as median (IQR) or n (%), unless otherwise indicated.
