# Supplementary material for: Clinical characteristics of pediatric patients hospitalized with community-acquired pneumonia and cytomegalovirus DNA detected in bronchoalveolar lavage fluid
Source: Front Pediatr. 2024 Jul 24;12:1407174. doi: 10.3389/fped.2024.1407174 (PMC11303221; doi:10.3389/fped.2024.1407174)
Supplement: Supplementary file 4 [file Table4.docx]

Table S4. Comparison of the clinical characteristics of patients with CMV replication for whom urine CMV testing was or was not performed.

| Parameter | Patients who underwent urine CMV testing (n=13) | Patients who did not undergo urine CMV testing (n=31) | *P* |
| --- | --- | --- | --- |
| General characteristics |  |  |  |
| Male | 12 (92.3) | 22 (71.0) | 0.123 |
| Age | 5.0 (2.0-8.5) | 5.0 (3.0-7.0) | 0.948 |
| Clinical signs and symptoms | |  |  |
| Fever | 7 (53.8) | 9 (29.0) | 0.118 |
| Wheezing | 10 (76.9) | 25 (80.6) | 0.780 |
| Disease severity |  |  |  |
| Requirement for supplemental oxygen | 6 (46.2) | 10 (32.3) | 0.382 |
| PICU admission | 4 (30.8) | 4 (12.9) | 0.161 |
| Mechanical ventilation | 3 (23.1) | 3 (9.7) | 0.237 |
| Laboratory findings |  |  |  |
| Peripheral leukocyte count, 10^9^/L | 13.8 (7.9-16.3) | 12.2 (9.0-17.7) | 0.990 |
| Neutrophil count, % | 34.9 (19.2-55.5) | 22.5 (14.4-37.1) | 0.169 |
| Hemoglobin, g/L | 113.0 (107.5-127.0) | 117.5 (107.0-126.5) | 0.731 |
| Platelet number 10^9^/L | 455.0 (365.5-568.5) | 415.0 (335.5-516.7) | 0.369 |
| C-reactive protein, mg/dL | 0.6 (0.05-6.4) | 0.3 (0.01-5.5) | 0.623 |
| Alanine transaminase, U/L | 32.0 (21.5-46.4) | 26.7 (20.0-35.2) | 0.297 |
| Aspartate aminotransferase, U/L | 46.8 (38.1-56.5) | 46.8 (36.7-57.2) | 0.969 |
| Bronchoalveolar lavage fluid cell profile | |  |  |
| Neutrophil, % | 45.0 (36.5-68.5) | 11.0 (1.2-61.0) | 0.410 |
| Alveolar macrophages, % | 50.0 (12.5-62.5) | 58.0 (25.7-89.2) | 0.291 |
| Lymphocytes, % | 3.0 (0.5-7.5) | 2.0 (0-6.7) | 0.601 |
| Eosinophils, % | 0 (0-0) | 0 (0-0) | 0.203 |

Data are presented as median (IQR) or n (%), unless otherwise indicated.
